# Osteopontin May Improve Postinjury Muscle Repair Via Matrix Metalloproteinases And tgf-β Activation in Regular Exercise

**DOI:** 10.7150/ijms.82925

**Published:** 2023-08-06

**Authors:** Yuchong Wang, Liang Hong, Jingjing Jiang, Xujun Zhang, Jianing Chen, Hongyan Diao

**Affiliations:** State Key Laboratory for Diagnosis and Treatment of Infectious Diseases, National Clinical Research Center for Infectious Diseases, National Medical Center for Infectious Diseases, Collaborative Innovation Center for Diagnosis and Treatment of Infectious Diseases, The First Affiliated Hospital, Zhejiang University School of Medicine, Hangzhou, 310003, China.

**Keywords:** Osteopontin, Muscle repair, Matrix metalloproteinases, TGF-β, Regular exercise

## Abstract

Skeletal muscle injuries are commonly observed during sports and trauma. Regular exercise promotes muscle repair; however, the underlying mechanisms require further investigation. In addition to exercise, osteopontin (OPN) contributes to skeletal muscle regeneration and fibrosis following injury. However, whether and how OPN affects matrix proteins to promote post-injury muscle repair remains uncertain. We recruited regular exercise (RE) and sedentary control (SC) groups to determine plasma OPN levels. Additionally, we developed a murine model of muscle contusion injury and compared the extent of damage, inflammatory state, and regeneration-related proteins in OPN knockout (OPN KO) and wild-type (WT) mice. Our results show that regular exercise induced the increase of OPN, matrix metalloproteinases (MMPs), and transforming growth factor-β (TGF-β) expression in plasma. Injured muscle fibers were repaired more slowly in OPN-KO mice than in WT mice. The expression levels of genes and proteins related to muscle regeneration were lower in OPN-KO mice after injury. OPN also promotes fibroblast proliferation, differentiation, and migration. Additionally, OPN upregulates MMP expression by activating TGF-β, which promotes muscle repair. OPN can improve post-injury muscle repair by activating MMPs and TGF-β pathways. It is upregulated by regular exercise. Our study provides a potential target for the treatment of muscle injuries and explains why regular physical exercise is beneficial for muscle repair.

## Introduction

Physical inactivity is associated with a high risk of various physical diseases including diabetes, coronary heart disease, and cancer. Some studies have reported that proper exercise enhances immunity and that people who regularly engage in exercise are often resistant to many diseases 1. Proper exercise contributes to the recovery of patients with muscular dystrophy, a primary muscle disease that results in skeletal muscle atrophy and weakness 2. Although high-intensity and excessive exercise can cause damage and inflammation in skeletal muscles, moderate- and endurance-intensity exercise increases muscle mass and plasticity 3-6. Muscle injuries compromise the health of the elderly population. During aging, the regenerative capacity of muscle stem cells decreases, diminishing the ability of muscles to repair following injury 7. Although it is widely accepted that exercise benefits health, the underlying mechanisms remain uncertain.

Skeletal muscle degeneration and regeneration involve well-characterized processes including myofiber necrosis, satellite cell activation and proliferation, myoblast migration and differentiation, and subsequent myofiber regeneration 8, 9. Remodeling of the extracellular matrix is beneficial for the migration of myoblasts to damaged sites for the fusion and regeneration of new muscle cells 8, 10. Additionally, evidence shows that the myogenic program activated by acute muscle injury and the subsequent inflammatory process are highly coordinated. In the early phase after acute injury, pro-inflammatory cytokines remove debris and regulate the proliferation, migration, and differentiation of satellite cells. Subsequently, anti-inflammatory cytokines attenuate inflammation and promote fibrosis repair 11. Exercise has been reported to increase the release of the anti-inflammatory cytokine interleukin-10, and encourages extracellular matrix remodeling to promote myofiber growth and repair 12, 13. However, the mechanism through which exercise affects skeletal muscle repair requires further investigation.

OPN is a multifunctional matricellular protein that is expressed in many cell types. Through cell-matrix and cell-cell interactions, OPN elicits a series of responses from numerous target cells via interactions with integrins and the hyaluronan receptor CD44. In many tissues, OPN is involved in important physiological and pathological processes, including wound healing, inflammation, and fibrosis 14-18. Previous studies have reported that OPN is generally undetectable in mature muscle fibers but is significantly elevated in muscles after injury 19, 20. Induction of OPN expression following skeletal muscle injury is associated with the infiltration of inflammatory cells, necrotic muscle fibers, and endogenous myogenic cells. Recently, several studies have demonstrated that neutrophil and macrophage infiltration, muscle fiber necrosis, and regeneration are delayed in the absence of OPN 21-23. OPN is highly expressed in the bone and contributes to osteogenesis. A previous study reported that moderate exercise increased bone OPN expression and reduced osteoporotic alterations in ovariectomized rats 24.

Thus, we hypothesized that OPN contributes to post-injury muscle repair and may be associated with moderate exercise. We recruited regular exercise (RE) and sedentary control (SC) groups to study the relationship between moderate exercise and OPN. We found that plasma levels of OPN and MMPs were elevated in the regular exercise group. We determined whether OPN plays an important role in muscle repair and regeneration. Our observations showed that OPN- deficiency mice exhibited delayed muscle fiber regeneration and repair after injury. We also found that OPN promoted the proliferation, differentiation, and migration of fibroblasts. Moreover, we suggest that OPN can improve post-injury muscle repair by activating the MMPs and TGF-β pathways. Regular exercise elevates the serum levels of OPN, TGF-β, and MMPs in mice, whereas OPN deficiency inhibits the expression of TGF-β and MMPs. In summary, we studied the relationship between OPN and postinjury muscle repair and discussed the health benefits of regular exercise. In particular, we determined how OPN affects matrix proteins and regulates myogenic and inflammatory processes to promote muscle repair.

## Materials and Methods

### Subjects

A total of 45 individuals were enrolled in the First Affiliated Hospital, School of Medicine, Zhejiang University, China, under the ethical approval of the Clinical Research Ethics Committee of the First Affiliated Hospital, College of Medicine, Zhejiang University, China (ref. 2020-110). Regular exercise (RE) group includes 20 college students in the badminton club (10 males and 10 females) who exercise three times a week. And 25 healthy sedentary college students (12 males and 13 females) were recruited as the sedentary control (SC) group (Table 1). Anticoagulant-treated blood samples were collected from participants after obtaining their informed consent.

### Animals

All animal experimental procedures were approved by the Animal Care and Use Committee of the First Affiliated Hospital, School of Medicine, Zhejiang University (reference number 2015‐186). The specific pathogen-free female mice and OPN-deficient mice (B6.129S6(Cg)-Spp1tm1Blh/J) were both of the same background (C57BL/6J) and both originally from Jackson Laboratory (Bar Harbor, ME, USA). 7-10 weeks old female wild-type (WT) mice and OPN-deficient (KO) mice were used to establish muscle injury model and exercise model.

### Muscle Contusion Injury

Contusion injury to a mouse hind-limb was produced using a mass-drop injury method similar as described in a previous study with slightly modification 26. Briefly, the technique entails dropping a 48.6-g weight from a height of 60 cm onto the medial surface of the gastrocnemius muscle after intraperitoneal injection with 1% pentobarbital. This contusion injury had a medium intensity and did not cause gait abnormalities. One of the mice's legs was injured and the other was used as a control.

### Exercise Studies

We used 7-week-old female mice type (WT) and OPN-deficient (KO) mice for the exercise studies. Mice in the RE group had free access to a running wheel for 2 h per day for 3 weeks 27.

### Histology and Immunohistochemistry

The injured muscles were cut to an appropriate size and fixed in 10% neutral buffered formalin for 1 d. For hematoxylin and eosin (H&E) staining, sections were rinsed with PBS and stained with hematoxylin for 40 min. The sections were subsequently rinsed under running tap water and stained with eosin for 10 min. For immunohistochemistry, slides were deparaffinized, treated with 3% H2O2 for 30 min, and blocked with 2% bovine serum albumin (BSA) for 60 min. The slides were incubated overnight at 4℃ with the primary rabbit monoclonal antibody (Abcam; ab32362; 1:1000 dilution). For antigen retrieval, sections were immunostained using the VECTASTAIN® ABC kit (UNIVERSAL, VECTOR, USA) following the manufacturer's specifications. Diaminobenzidine (DAB) was used for staining, and the sections were counterstained with hematoxylin.

### ELISA

The plasma was separated from blood samples by centrifugation at 3000 g for 10 min at room temperature. The plasma levels of OPN, TGF-β1, TGF-β2 and TGF-β3 were detected by ELISA kits (eBioscience, San Diego, CA, USA) following the manufacturer's instructions. The level of MMP2, MMP3, MMP7, MMP9, MMP12, MMP13 were detected by ELISA kits (Biotechnology Systems).

### Analysis of mRNA expression

Total RNA was isolated using the TRIzol reagent (Life Technologies, Gaithersburg, MD, USA) according to the manufacturer's instructions. cDNA was synthesized using a HiScript II 1st Strand cDNA Synthesis Kit (Vazyme, China). Relative gene expression was analyzed according to the instructions for the SYBR Premix Ex Taq kit ChamQ Universal SYBR qPCR Master Mix (Vazyme, China). The levels of fibronectin (FN), tenascin-C (TN-C), and TGF-β1 mRNA expression in the different groups were determined by real-time quantitative PCR (qPCR) using an ABI Prism 7000 Sequence Detection System (Applied Biosystems Life Technologies, Foster City, CA, USA) with GAPDH as an internal standard. The specific primers used were as follows: GAPDH, 5'-ATC CCA TCA CCA TCT TCC AGG-3' (sense), 5'-GAG CCC CAG CCT TCT CCATG-3' (anti-sense); TN-C, 5′-TTT GCC CTC ACT CCC GAAG-3′ (sense), 5′-AGG GTC ATG TTT AGC CCA CTC-3′ (anti-sense); FN, 5'-ATG TGG ACC CCT CCT GAT AGT-3' (sense), 5'-GCC CAG TGA TTT CAG CAA AGG-3' (anti-sense); TGF-β1, 5'- CTC CCG TGG CTT CTA GTGC-3' (sense), 5'- GCC TTA GTT TGG ACA GGA TCTG-3' (anti-sense). Values for FN, TN-C and TGF-β1 were normalized against those for GAPDH.

### Immunofluorescence

For immunofluorescence staining, sections were fixed with 4% paraformaldehyde and blocked for 1 h. Sections were incubated overnight at 4℃ with indicated primary antibodies (Abcam; ab52866; 1:250 dilution) diluted in blocking buffer. Subsequently, sections were incubated with secondary antibodies and DAPI at room temperature for 50 min.

### Cell culture and treatment

Immortalized cell lines NIH/3T3 was cultured in Dulbecco's modification of Eagle's medium Dulbecco (DMEM) supplemented with 10% fetal bovine serum (FBS) and 1% penicillin-streptomycin solution at 37℃, 5% CO2 cell incubator. NIH/3T3 cells were cultured with recombinant OPN (100ng/ml; MedChemExpress) for subsequent experiments.

### Statistical analysis

Data are presented as mean ± SD and are representative of at least two independent experiments *in vitro*. The unpaired Student's t-test test and one-way analyses of variance (ANOVA) were used for statistical analysis. Statistical significance was set at P < 0.05.

## Results

### Regular Exercise Induces the Increase of OPN and MMPs in Peripheral Blood

**Figure 1:**
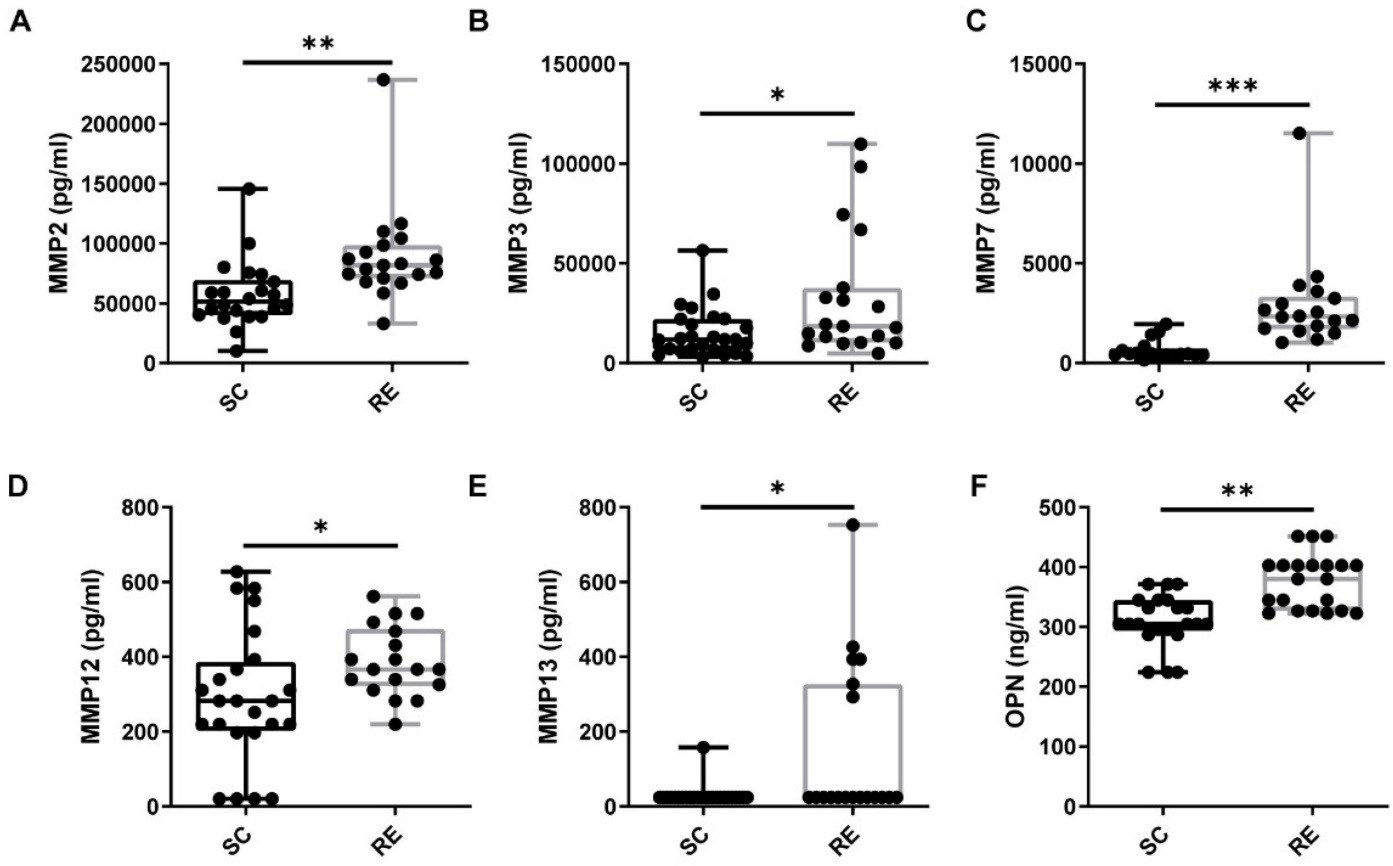
** Regular Exercise Induces the Increase of OPN and MMPs Expression in Plasma. (A)** People in the regular exercise (RE) group showed increased OPN expression in plasma than sedentary control (SC).** (B-F)** MMP2, MMP3, MMP7, MMP12, and MMP13 levels in plasma were upregulated in the RE group. The results are expressed as means ± range. *p < 0.05, determined by two-tailed Student's t-test.

Regular physical exercise contributes to postinjury muscle repair. To study the expression levels of cytokines related to muscle remodeling in individuals who exercise regularly and to determine whether OPN is involved, we first determined the plasma levels of OPN in the RE and SC groups. We found that the plasma expression of OPN was significantly higher in the RE group than in the SC group (Figure 1A). These findings supported the hypothesis that OPN modulates exercise-induced immunity. As we hypothesized that OPN affects some matrix proteins that promote muscle repair, we next determined the plasma MMP levels in the RE and SC groups. These are essential for muscle remodeling after injury. MMPs can degrade the extracellular matrix, thus helping cells move for rearrangement and allowing muscle satellite cells to migrate during muscle remodeling 28-30. In line with our hypothesis, significant increases occurred in the plasma levels of MMPs in the RE group compared to those in the SC group (Figure 1B-F). Notably, among the matrix metalloproteinases we detected, MMP2 was remarkably upregulated. This was frequently reported to be associated with muscle regeneration.

### OPN improved Muscle Fiber Regeneration

**Figure 2:**
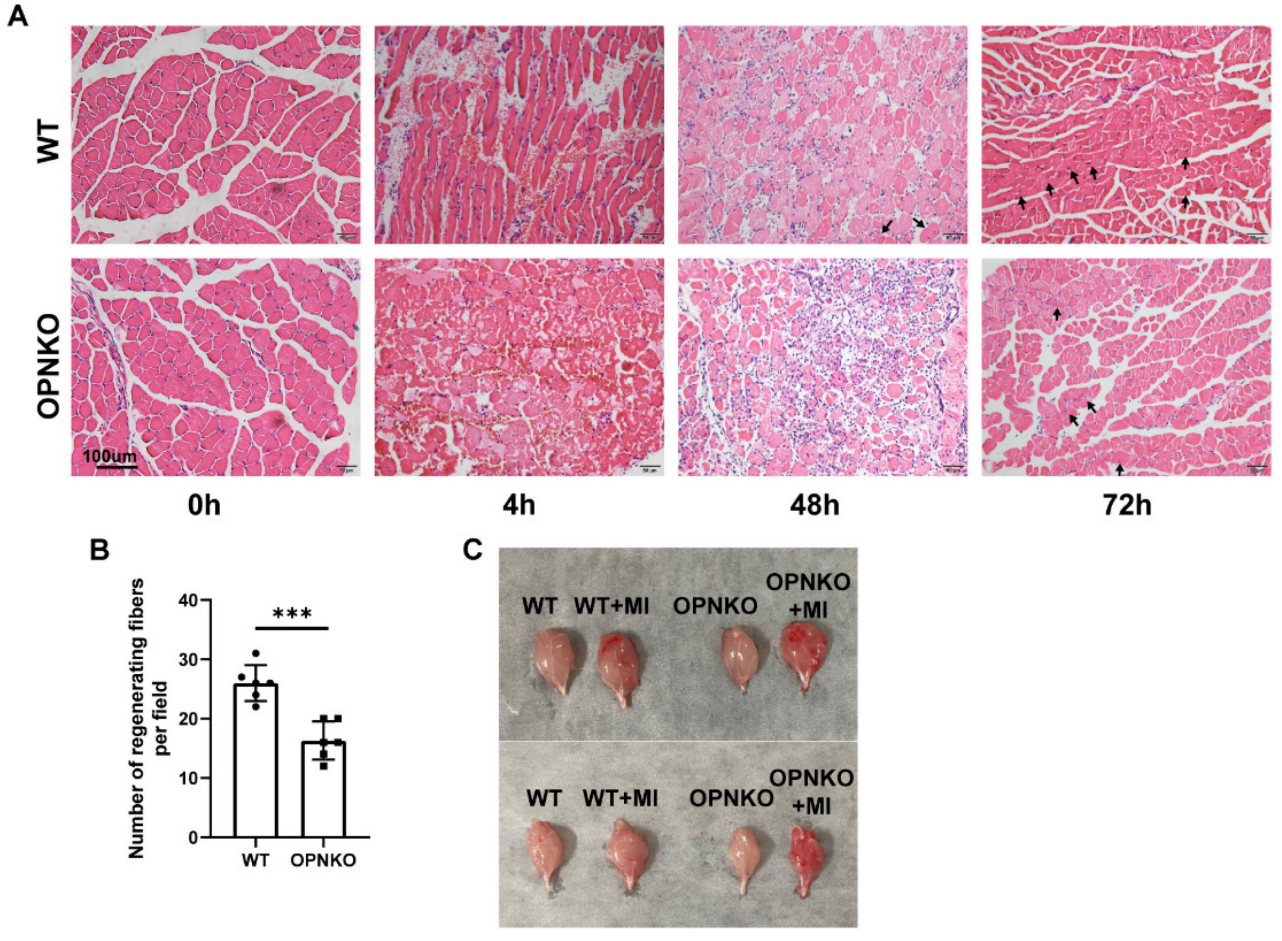
** OPN Promoted Muscle Repair after Contusion Injury. (A)**H&E staining of the gastrocnemius muscle of the OPN-KO and WT mice before and 4, 48, and 72 h after contusion-induced muscle injury. Arrows indicate regenerating muscle fibers (centrally placed nuclei). Scale bar = 100 µm. **(B)** Quantification of regenerating muscle fibers at 72 hours post-injury. **(C)** Gastrocnemius muscle of the OPN-KO and WT mice in the figure before or 48h after muscle injury (MI). The mice were randomly assigned to four groups (6-10 mice/group). Data were analyzed using a two-tailed Student's t-test. Values are presented as mean ± SD, *p < 0.05.

To verify the promoting effect of OPN on muscle repair after injury, we generated a reproducible mouse model that recapitulated the significant manifestations of such an injury, including nociceptive sensitization, muscle disruption, and muscle fiber loss, without bone fracture. Based on previous studies, we used a mouse model for gastrocnemius contusion injury. As expected, the OPN-KO mice showed worse histological manifestations, such as red blood cell accumulation, inflammatory cell infiltration, and fewer regenerated muscle fibers than the WT mice at 48 and 72 h after injury, despite no obvious differences at 4 h after injury (Figure 2A-B). The gastrocnemius muscle isolated from OPN-KO mice after injury was congestive and in a more inflammatory or injured state compared to WT mice (Figure 2C). These results support our hypothesis that OPN accelerates postinjury muscle repair. Moreover, our findings indicate that OPN deficiency affects the process of muscle repair rather than the severity of the damage.

**Figure 3:**
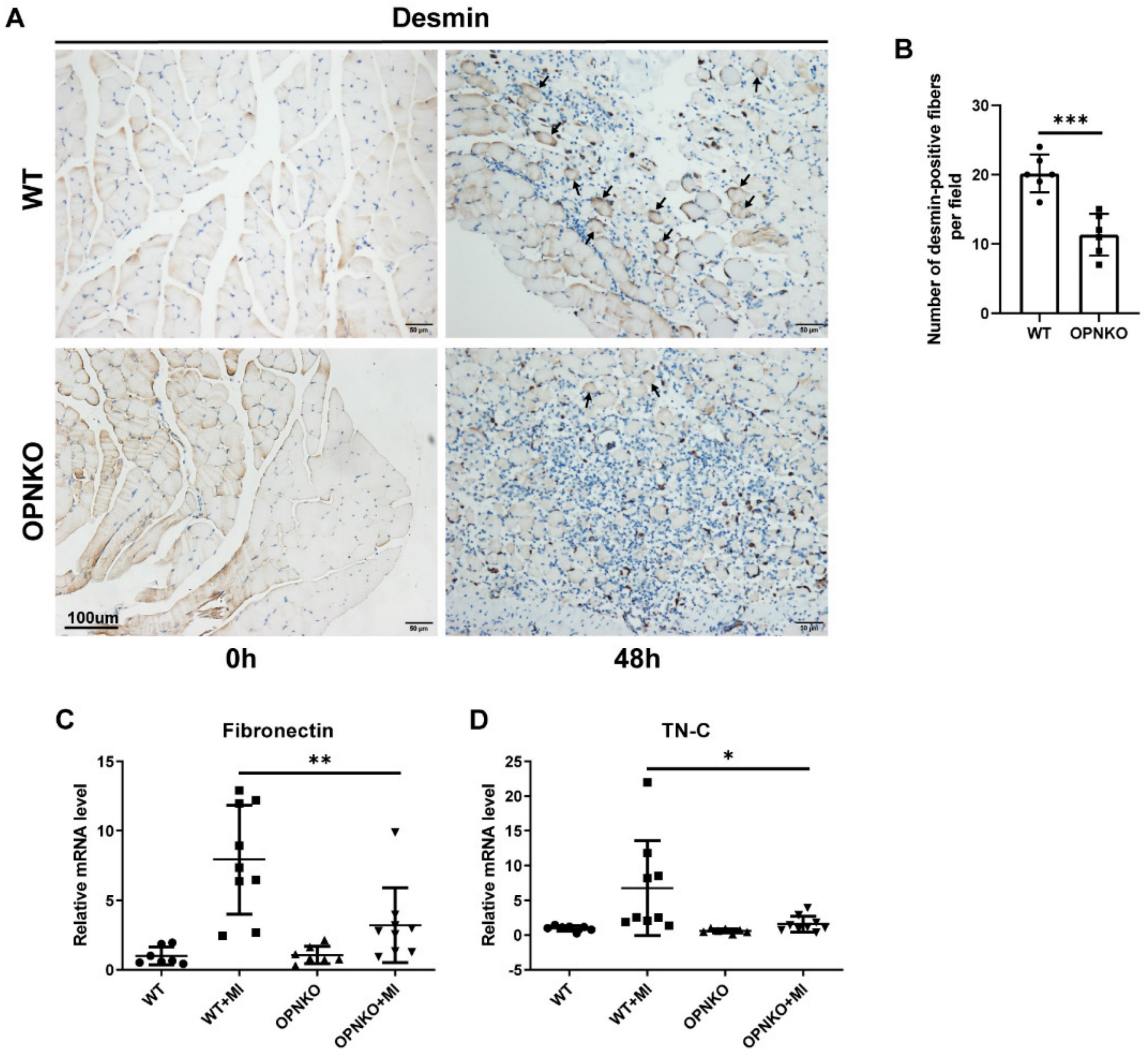
** OPN Improved Muscle Fiber Regeneration. (A)** IHC staining of desmin protein in muscle tissue before and after injury. Arrows indicate desmin-positive fibers. Scale bar = 100 µm. **(B)** Quantification of desmin-positive fibers at 48 hours post-injury. **(C-D)** qPCR analysis of fibronectin and tenascin-C (TN-C) in OPN-KO and WT mice before and 48h after muscle injury respectively. Data were analyzed using a two-tailed Student's t-test or one-way ANOVA. Values are mean ± SD, *p < 0.05.

Next, we evaluated the regeneration of the gastrocnemius muscle fibers and found that the OPNKO group exhibited delayed muscle regeneration. A desmin indicator was used to evaluate muscle fibers regeneration. The expression of desmin in the contusion-injured muscles of OPN-KO mice exceeded that in WT mice (Figure 3A-B). These results indicate that OPN deficiency can reduce desmin expression during contusion, which consequently can inhibit the regeneration of muscle fibers. The FN is a component of the extracellular matrix. FN can regulate fibroblast growth factor (FGFs) signaling, which is essential for the self-renewal of muscle satellite cells and affects the regenerative capacity of skeletal muscles in mice 31, 32. It has also been reported that FN promotes directional collective migratory behavior, favoring cell-cell alignment and fusion 33. In homeostatic tissues, TN-C is restricted to areas of high loading, such as tendons and myotendinous junctions. It is hypothesized to enhance mechanical stability. After muscle injury, TN-C is upregulated in injured and regenerating skeletal muscles and is thought to decrease cell adhesion, promote migration, and inhibit premature fusion 34, 35. We next detected the expression levels of FN and TN-C using the qPCR analysis to evaluate muscle regeneration and found that their expression was reduced in the OPN-KO group compared to that in the WT group (Figure 3C-D).

### OPN Accelerated the Progress of Fibrosis Repair

**Figure 4:**
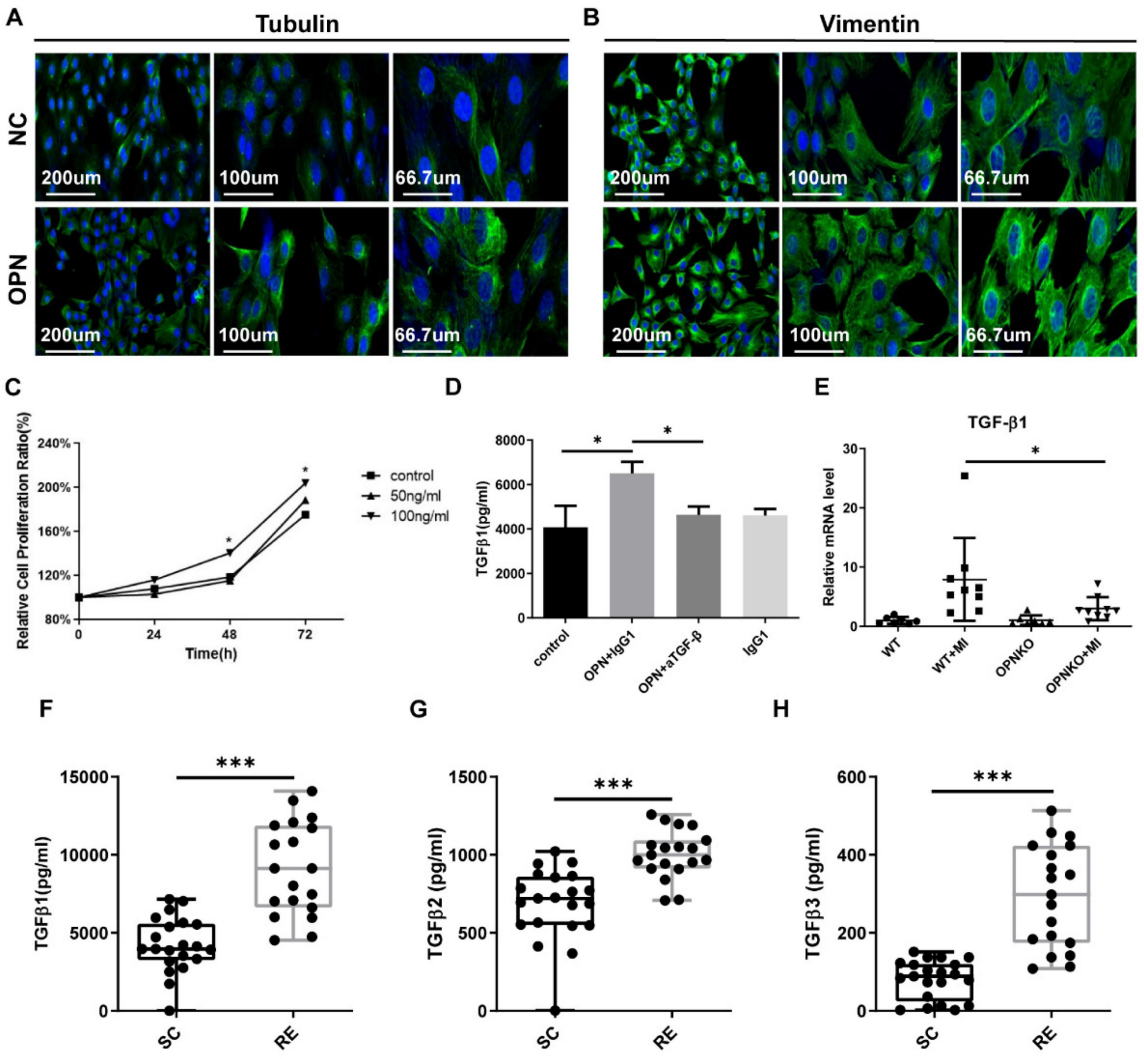
** Myofibroblasts Got Activated after OPN Cultivation and TGF-β Expression Was Enhanced. (A-B)** Myofibroblasts were cultured *in vitro* and stained 72 h after OPN cultivation (DAPI=blue, tubulin, or vimentin=green). Fluorescence micrographs were pictured under different multiples. **(C)** The proliferation of myofibroblasts cultured with OPN for 48h, and 72h with a concentration of 100ng/ml was promoted. Data were analyzed with one-way ANOVA. **(D)** OPN promoted the secretion of TGF-β1 in myofibroblasts, and this effect was blocked by TGF-β inhibitors. This was determined by a two-tailed Student's t-test. **(E)** qPCR analysis of TGF-β1 in OPN-KO and WT mice before and 48h after muscle injury. Data were analyzed with one-way ANOVA. Values are mean ± SD, *p < 0.05. **(F-H)** Plasma levels of the TGF-β family were elevated in the RE group compared to the SC group. The results are expressed as means ± range. *p < 0.05, determined by two-tailed Student's t-test.

Evidence shows that the myogenic program activated by acute muscle injury and the subsequent inflammatory process are highly coordinated. In the early phase after acute injury, pro-inflammatory cytokines remove debris and regulate the proliferation, migration, and differentiation of satellite cells. Anti-inflammatory cytokines attenuate inflammation and promote repair 18. It is generally accepted that fibroblast-to-myofibroblast differentiation represents a key event during wound healing and tissue repair. Thus, we subsequently investigated whether fibroblasts were activated by OPN. We found that OPN treatment facilitated the proliferation of fibroblasts (Figure 4A). Hence, we increased the cytoskeleton formation (Figure 4B-C). The cytoskeletal proteins vimentin and tubulin are essential for cell migration and mediation of myofibroblast differentiation. Vimentin is critical for the function of repair cells and is a director of the wound healing process. Microtubules such as tubulin are involved in the extension of vimentin filaments in repair cells 36-38. TGF-β1 has important regulatory effects on cell growth, fibroblast-to-myofibroblast differentiation, and immune function. As an anti-inflammatory cytokine, TGF-β can attenuate inflammation and promote the repair of fibrosis. We found that OPN increased the secretion of TGF-β1 (Figure 4D). Additionally, we discovered that the expression of TGF-β1 in the injured muscles in the OPN-KO group was lower than that in the WT group (Figure 4E). These data indicate that the OPN can activate fibroblasts and TGF-β signaling after muscle injury. This is beneficial for muscle repair. We also found that the plasma levels of TGF-β in the RE group were significantly elevated than the SC group (Figure 4F-H). To some extent, we can hypothesize that individuals who show upregulation of OPN and TGF-β caused by regular exercise may have better healing abilities than sedentary individuals.

### MMPs Were Upregulated by OPN Via TGF-β Signaling

**Figure 5:**
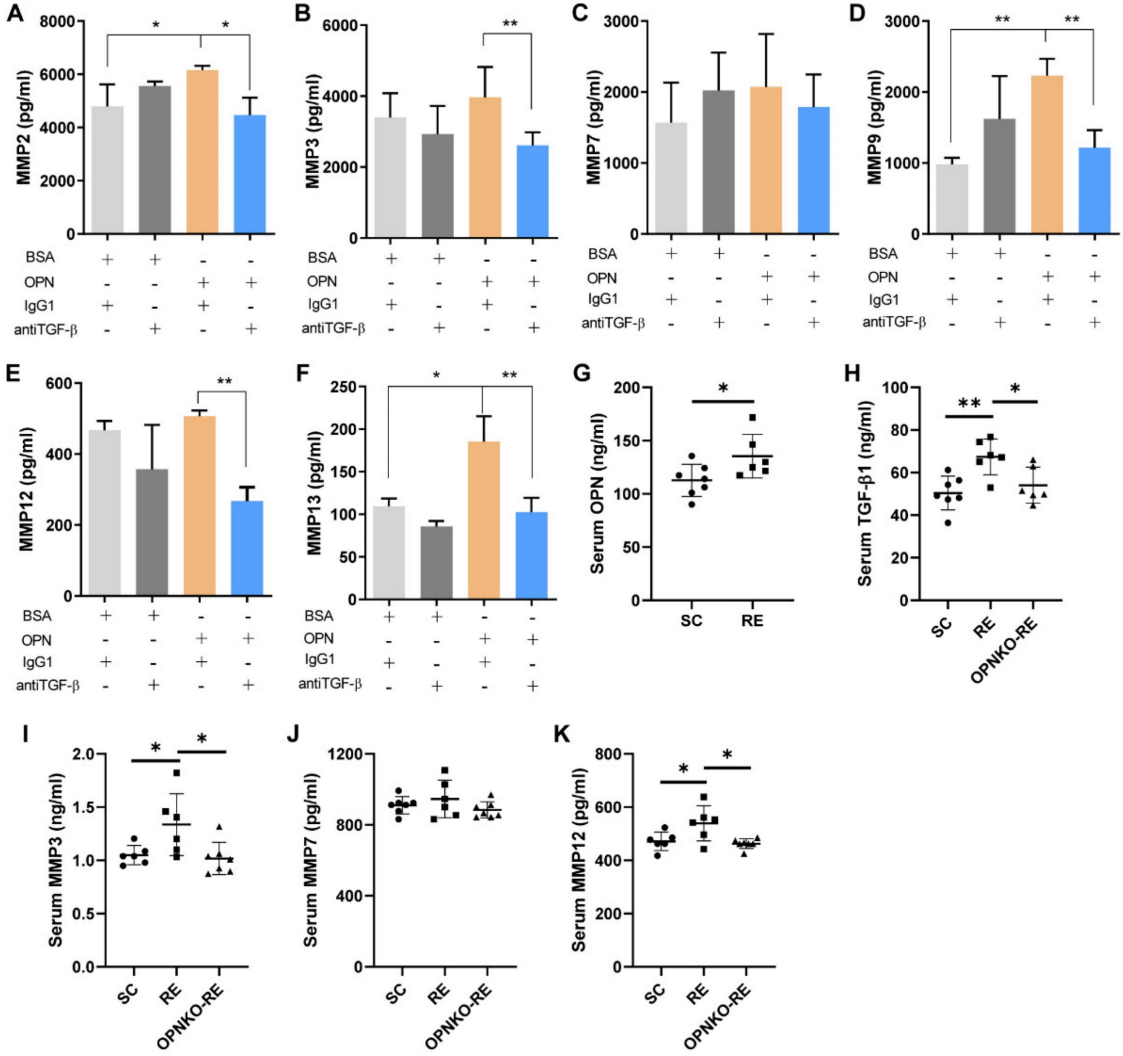
** MMPs Were Upregulated by OPN through TGF-β Signaling. (A-F)** The levels of MMP2, MMP3, MMP7, MMP9, MMP12, and MMP13 were increased after OPN cultivation, which was blocked by TGF-β inhibitors. **(G)** Serum levels of OPN in RE and SC group of mice. **(H-K)** Serum levels of TGF-β1, MMP3, MMP7, and MMP12 in SC, RE, and OPNKO-RE group of mice. 6-10 mice/group. Results are expressed as means ± SD. *p < 0.05, determined by two-tailed Student's t-test.

Because the expression levels of MMPs were elevated in the RE group, we aimed to determine whether OPN promoted muscle repair by activating MMPs. Previous data demonstrated that TGF-β was involved in the pathway through which OPN affects muscle repair. Thus, we hypothesized that TGF-β contributes to MMPs activation. We found that myofibroblasts cultivated by OPN expressed increased levels of MMPs, but the levels were not increased when added with TGF-β inhibitors (Figure 5A-F). In the RE group, we found increased serum levels of OPN, TGF-β1, MMP3, and MMP12 compared to those in the SC group (Figure 5G-K). These results are consistent with those of a previous clinical RE study. Furthermore, we measured serum levels of TGF-β and MMPs in OPNKO-RE mice. The results showed that OPNKO-RE mice had decreased levels of TGF-β1, MMP3, and MMP12 than WT-RE mice (Figure 5H-K). These results suggested that OPN activates MMPs via TGF-β signaling.

## Discussion

Injury to skeletal muscles, especially mechanically induced damage, such as contusion injury, frequently occurs in contact sports. The recovery phase is characterized by inflammation, muscle precursor cell activation and growth, and muscle fiber repair. The severity of various injuries and inflammatory responses complicate the identification of suitable treatments. To increase the chances of successful treatment, it is important to understand the mechanisms underlying skeletal muscle injury and the cellular processes involved in muscle healing following a contusion injury. Although excessive inflammation is thought to be harmful to recovery, appropriate degrees of inflammation can accelerate the resolution process and promote muscle regeneration and fiber repair. For example, macrophages, which form part of the inflammatory response, can remove cellular debris and secrete cytokines and growth factors to facilitate muscle regeneration and fiber repair. Rather than exacerbating secondary damage, it plays a role in the recovery 39. OPN is a pro-inflammatory factor that increases significantly after muscle injury 19, 20. In addition, OPN promotes fibrosis and inflammation in multiple diseases, particularly chronic liver disease. These findings led us to hypothesize that OPN may promote muscle repair after injury. Several studies have suggested that OPN deficiency can cause a delay in neutrophil and macrophage infiltration, muscle fiber necrosis, and regeneration after muscle injury 21-23. We further verified these findings and elucidated their underlying mechanisms.

Moderate and regular exercise are considered beneficial for recovery from trauma. These mechanisms vary and involve a wide range of immune fields. To discuss more preventive and feasible treatments, we considered regular exercise as an approach to obtain a stronger recovery ability. We attempted to link OPN expression to regular exercise because both can improve muscle repair. Herein, we found that the plasma levels of OPN were significantly elevated in the serum of moderate- and regular-exercise participants.

Muscle regeneration requires the coordination of several factors to mobilize satellite cells, remodel the extracellular matrix surrounding the muscle fibers, and repair existing and/or form new muscle fibers. MMPs have been reported to contribute to skeletal muscle regeneration, growth, myoblast migration, and vascular tissue remodeling. MMPs are often regulated by endogenous tissue inhibitors of metalloproteinases (TIMPs), and the MMP/TIMP ratio often determines the extent of extracellular matrix (ECM) protein degradation and tissue remodeling 40-42. Degradation and remodeling of the ECM are beneficial for the migration of myoblasts to damaged sites for the fusion and regeneration of new muscle cells. As a matricellular protein, OPN influences extracellular matrix remodeling and is linked to inflammation and myogenesis during the regeneration of injured skeletal muscles 11. Therefore, we investigated whether OPN regulated muscle repair and regeneration via MMPs. Recent studies have also reported that the TGF-β family is involved in muscle remodeling, where evidence is emerging that TGF-β proteins act with myostatin to regulate the growth and remodeling of skeletal muscle. The dysregulation of TGF-β proteins and their associated signaling components is increasingly implicated in muscle wasting associated with chronic illness, aging, and inactivity 43. Our results showed that regular exercise increased MMPs and TGF-β family expression in human plasma and mouse serum. Moreover, our observations showed that OPN significantly enhanced TGF-β signaling both in myofibroblasts and injured muscle tissue. Additionally, OPN upregulated the expression of MMPs via activating TGF-β, which indicates that OPN can improve post-injury muscle repair by activating MMPs and TGF-β pathways. Moreover, it is upregulated by regular exercise.

In this study, we demonstrated the essential role of OPN in skeletal muscle repair associated with regular exercise. Studies have reported that moderate endurance exercise increases muscle mass and plasticity and that resistance exercise is related to muscle recovery and remodeling 3-6. OPN influences extracellular matrix remodeling and is associated with inflammation and myogenesis. Therefore, we investigated the specific regulatory mechanisms of OPN during postinjury muscle repair. This observation demonstrates that OPN is essential for skeletal muscle repair, including regeneration and fibrosis. *In vitro*, OPN promoted the proliferation, differentiation, and migration of fibroblasts. In particular, we demonstrated that OPN may regulate muscle repair by activating TGF-β/MMPs. Owing to some limitations, we did not study whether the increase in OPN and MMPs caused by regular exercise was effective for muscle regeneration in mice. These will be replenished in the future.

To our knowledge, current studies have not discussed the relationship among OPN, MMPs, and TGF-β. We have reported for the first time the changes in OPN, MMPs, and TGF-β plasma expression in people who exercise regularly and demonstrated for the first time that OPN can upregulate MMPs expression by TGF-β activation. Collectively, our results provide evidence that OPN may upregulate MMPs to benefit postinjury muscle repair through the TGF-β pathway. Because the plasma expression of OPN, MMPs, and TGF-β in regular exercise people is also elevated, we can conjecture that people who exercise regularly may get better healing abilities. Our findings provide cellular evidence for the protective role of OPN in muscle repair, leading to strategies for the treatment of skeletal muscle injury.

## Figures and Tables

**Table 1:** Clinical characteristics of the participants in the two groups 25.

	SC (n=25)	RE (n=20)	p value
Age (years)	21.8 ± 1.5	20.8 ± 2.1	0.05689
Males (%)	12 (48%)	10 (50%)	0.8676

## Abbreviations

ELISA: Enzyme-linked immunosorbent assay
FN: Fibronectin
H&E: Hematoxylin and eosin
MMPs: Matrix metalloproteinases
OPN: Osteopontin
OPNKO: Osteopontin knock-out
qPCR: Quantitative polymerase chain reaction
RE: Regular exercise
SC: Sedentary control
TGF-β: Transforming growth factor-β
TN-C: Tenascin-C
TIMPs: Tissue inhibitors of metalloproteinases
WT: Wild-type
